# Mitochondrial genetic variation and risk of chronic kidney disease and acute kidney injury in UK Biobank participants

**DOI:** 10.1007/s00439-023-02615-4

**Published:** 2024-02-13

**Authors:** Vasantha Jotwani, Stephanie Y. Yang, Heather Thiessen-Philbrook, Chirag R. Parikh, Ronit Katz, Gregory J. Tranah, Joachim H. Ix, Steve Cummings, Sushrut S. Waikar, Michael G. Shlipak, Mark J. Sarnak, Samir M. Parikh, Dan E. Arking

**Affiliations:** 1 Department of Medicine, Kidney Health Research Collaborative, San Francisco Veterans Affairs Health Care System and University of California San Francisco, 4150 Clement Street, Bldg 2, Rm 145, San Francisco, CA 94121 USA; 2 Department of Genetic Medicine, McKusick-Nathans Institute, Johns Hopkins University School of Medicine, Baltimore, MD USA; 3 Division of Nephrology, School of Medicine, Johns Hopkins University, Baltimore, MD USA; 4 Department of Obstetrics and Gynecology, University of Washington, Seattle, WA USA; 5 California Pacific Medical Center Research Institute, San Francisco, CA USA; 6 Division of Nephrology-Hypertension, University of California San Diego, and Veterans Affairs San Diego Healthcare System, San Diego, CA USA; 7 Section of Nephrology, Department of Medicine, Boston University School of Medicine and Boston Medical Center, Boston, MA USA; 8 Division of Nephrology, Tufts Medical Center, Boston, MA USA; 9 Division of Nephrology, Department of Medicine, and Department of Pharmacology, University of Texas Southwestern Medical School, Dallas, TX USA

## Abstract

Experimental models suggest an important role for mitochondrial dysfunction in the pathogenesis of chronic kidney disease (CKD) and acute kidney injury (AKI), but little is known regarding the impact of common mitochondrial genetic variation on kidney health. We sought to evaluate associations of inherited mitochondrial DNA (mtDNA) variation with risk of CKD and AKI in a large population-based cohort. We categorized UK Biobank participants who self-identified as white into eight distinct mtDNA haplotypes, which were previously identified based on their associations with phenotypes associated with mitochondrial DNA copy number, a measure of mitochondrial function. We used linear and logistic regression models to evaluate associations of these mtDNA haplotypes with estimated glomerular filtration rate by serum creatinine and cystatin C (eGFR_Cr-CysC_, *N* = 362,802), prevalent (*N* = 416 cases) and incident (*N* = 405 cases) end-stage kidney disease (ESKD), AKI defined by diagnostic codes (*N* = 14,170 cases), and urine albumin/creatinine ratio (ACR, *N* = 114,662). The mean age was 57 ± 8 years and the mean eGFR was 90 ± 14 ml/min/1.73 m^2^. MtDNA haplotype was significantly associated with eGFR (*p* = 2.8E−12), but not with prevalent ESKD (*p* = 5.9E−2), incident ESKD (*p* = 0.93), AKI (*p* = 0.26), or urine ACR (*p* = 0.54). The association of mtDNA haplotype with eGFR remained significant after adjustment for diabetes mellitus and hypertension (*p* = 1.2E−10). When compared to the reference haplotype, mtDNA haplotypes I (*β* = 0.402, standard error (SE) = 0.111; *p* = 2.7E−4), IV (*β* = 0.430, SE = 0.073; *p *= 4.2E−9), and V (*β* = 0.233, SE = 0.050; *p* = 2.7E−6) were each associated with higher eGFR. Among self-identified white UK Biobank participants, mtDNA haplotype was associated with eGFR, but not with ESKD, AKI or albuminuria.

**Supplementary Information:**

The online version contains supplementary material available at 10.1007/s00439-023-02615-4.

## Introduction

Chronic kidney disease (CKD) and acute kidney injury (AKI) are major public health problems with tremendous impact on morbidity and mortality worldwide (Rhee and Kovesdy 2015). Mitochondrial dysfunction has been implicated in the pathogenesis of CKD and AKI due to the roles of mitochondria in oxidative phosphorylation, reactive oxygen species generation, and programmed cell death (Che et al. 2014; Eirin et al. 2014; Small et al. 2012; Hall and Unwin 2007; Tran et al. 2011, 2016). Within the kidney, mitochondria are particularly important for the tubules, where oxygen consumption by mitochondria is driven by the need for active solute transport (Weinberg et al. 2000; Hansell et al. 2013).

Mammalian mitochondria possess a maternally inherited, intronless 16,569 base-pair genome (mtDNA) containing 37 genes that encode polypeptides, tRNAs, and rRNAs (Chinnery and Hudson 2013; Taanman 1999). The majority of these genes are essential for the machinery that converts metabolic energy into adenosine triphosphate. Recent studies within the UK Biobank and the Biobank Japan Project reported associations of mtDNA variants with kidney function, assessed by estimated glomerular filtration rate (eGFR) (Yonova-Doing et al. 2021; Yamamoto et al. 2020). Specific mtDNA variants have also been associated with tubulointerstitial kidney disease, IgA nephropathy, and focal segmental glomerulosclerosis (Douglas et al. 2014; Connor et al. 2017; Doleris et al. 2000; Hotta et al. 2001). To our knowledge, no prior study has examined associations of mitochondrial genetic variants with the risk of ESKD, AKI or albuminuria.

We previously assessed the impact of mitochondrial genetic variation on mitochondrial DNA copy number (mtDNA-CN), a measure of mitochondrial abundance, along with 41 additional traits associated with mtDNA-CN within the UK Biobank Study (Longchamps et al. 2022). This prior analysis identified six mtDNA single nucleotide polymorphisms (mtSNPs) with independent effects that correspond to eight haplotypes with minor allele frequency > 0.005. Given the haploid nature of the mitochondrial genome, haplotypes can capture potential synergistic or compensatory effects between mtSNPs. In this study, we investigated associations of these haplotypes with eGFR, end-stage kidney disease (ESKD) risk, AKI risk, and albuminuria in participants of the UK Biobank. Among the subset of participants with whole-genome sequencing data, we also evaluated associations of common mtSNPs with the kidney outcomes of interest.

## Materials and methods

### Study design and population

The UK Biobank is a large-scale cohort with medical and genetic data collected from 502,419 participants residing in the United Kingdom (Bycroft et al. 2018). Briefly, participants aged 37–73 years of age were recruited from 22 centers between 2006 and 2010. The assessment visit included a standardized questionnaire with detailed sociodemographic, lifestyle and medical information, a physical examination, and collection of biological specimens. The database enables linkage to a range of health-related records and outcomes, including data obtained from hospitalizations and from national death and cancer registries. The present study was conducted in accordance with the Declaration of Helsinki. Ethical approval for the UK Biobank was provided by the National Health Service National Research Ethics Service (21/NW/0157). All participants provided written informed consent for data use in research studies.

The present study included all unrelated (“used.in.pca.calculation = 1”) self-identified white participants with mtDNA genotyping data available and without prevalent end-stage renal disease. Other racial and ethnic groups were excluded from the analysis due to their small sample sizes. Among these 381,039 participants, eight distinct mtDNA haplotypes accounted for 379,432 participants. At the assessment visit, serum creatinine and cystatin C measurements were available for estimation of glomerular filtration rate in 362,802 participants, and albuminuria measurements were available in 114,662 participants.

### Predictors

MtDNA haplotypes were identified within the UK Biobank based on association studies performed with approximately 150 mtSNPs (minor allele frequency, MAF > 0.5%) with mtDNA-CN and 41 additional traits as described previously (Longchamps et al. 2022). Briefly, mtDNA genotype phasing and imputation were performed using the 1000 Genomes Project phase 3 mtDNA variants as the reference panel (Genomes Project C et al. 2015). The SusieR package was used to identify potential causal variants for each independent locus associated with the outcomes of interest, and resulted in a total of six independent mtSNPs across four traits (MT73, MT7028, MT10238, MT12612, MT13617, MT15257) (Wang et al. 2020). Haplotypes were constructed by concatenating the six SNPs, resulting in eight haplotypes with minor allele frequency > 0.5%, with the most prevalent haplotype set as the reference (Supplemental Table 1). The primary analysis focused on using haplotypes to minimize the multiple-testing burden from a single SNP approach and to account for multiple functional variants within an individual. Secondary analyses were performed using whole-genome sequencing data which were available from 144,741/381,039 participants.

### Outcomes

Estimated glomerular filtration rate (eGFR) was calculated by the 2012 CKD-EPI equation for serum creatinine and cystatin C (eGFR_Cr-CysC_) (Inker et al. 2012). In sensitivity analyses, we calculated eGFR by the 2012 CKD-EPI equation for serum cystatin C (eGFR_CysC_) alone. We analyzed eGFR as a continuous outcome and as a dichotomous outcome, eGFR < 60 ml/min/1.73 m^2^. Prevalent and incident ESKD events were ascertained algorithmically as defined previously by UK Biobank (UK Biobank Outcome Adjudication Group 2022) and included individuals with Stage 5 CKD who received renal replacement therapy and those who received a kidney transplant. The AKI outcome was defined by ICD9 and ICD10 codes (Supplemental Table 2) and included past and incident events. Albuminuria was quantified as the albumin-to-creatinine ratio (ACR, mg/g) in spot urine specimens collected at the assessment visit, and was evaluated as a continuous outcome and as a dichotomous outcome, ACR > 30 mg/g. Participants with ESKD at baseline were excluded from analyses of eGFR, AKI, and ACR.

### Covariates

Covariates for adjustment included age, sex, center, genotyping principal components, and genotyping array (Bycroft et al. 2018). Diabetes mellitus was defined by any one of the following: self-report, ICD9 or ICD10 diagnostic codes (Supplemental Table 2), insulin use, or hemoglobin A1c > = 48 mmol/mol. Hypertension was defined by any one of the following: antihypertensive medication use, systolic blood pressure > = 140 mm Hg, or diastolic blood pressure > = 90 mm Hg.

### Statistical analysis

Baseline characteristics were compared across mtDNA haplotypes. Linear and logistic regression analyses were used to evaluate associations of mtDNA haplotypes and mtSNPs with the outcomes of interest. Individuals with missing data were excluded. To explore the potential effects of ancestry-related haplogroups on associations of haplotype with eGFR, we conducted regression analyses with and without haplogroup in the model. Due to its right-skewed distribution, urine ACR was log-transformed for analyses. Statistical significance was evaluated by ANOVA between regression models with and without the haplotypes, using the most prevalent haplotype as the reference group. Primary analyses were adjusted for age, age squared, sex, center, 40 principal components, and genotyping array. In exploratory analyses, we adjusted for diabetes mellitus and hypertension as potential mediators. Cox proportional hazards models were used to evaluate associations of mtDNA haplotypes with the outcome of incident ESKD. To account for multiple testing with five independent tests (eGFR, prevalent and incident ESKD, AKI and ACR), we used a Bonferroni corrected *p* value < 0.01 as the study-wide threshold for statistical significance. All analyses were performed using R version 4.0.3.

## Results

### MtDNA haplotypes and baseline characteristics of UK Biobank participants

Among the 381,039 participants with available mtDNA genotyping data, eight distinct mtDNA haplotypes accounted for 379,432 participants (99.6%). The most prevalent mtDNA haplotypes accounted for 40% and 28% of participants, respectively (Table 1). The remaining mtDNA haplotypes were much less common, each accounting for fewer than 10% of participants.

**Table 1:** Baseline characteristics of UK Biobank participants overall and by mtDNA haplotype

	Overall	mtDNA haplotype							
		Reference	I	II	III	IV	V	VI	VII
*N* (%)	379,432 (100)	150,790 (40)	13,467 (4)	6892 (2)	34,453 (9)	34,801 (9)	104,630 (28)	14,450 (4)	19,949 (5)
Age (y)	56.7 (8)	56.7 (8)	56.7 (8)	56.8 (8)	56.8 (8)	56.7 (8)	56.7 (8)	56.7 (8)	56.7 (8)
Female	205,026 (54)	81,473 (54)	7263 (54)	3710 (54)	18,763 (54)	18,872 (54)	56,406 (54)	7720 (54)	10,819 (54)
Diabetes mellitus	21,006 (6)	8363 (6)	769 (6)	367 (5)	2006 (6)	1853 (5)	5752 (6)	768 (5)	1128 (6)
Hypertension	194,867 (54)	77,600 (54)	6804 (53)	3559 (54)	17,478 (54)	17,820 (54)	53,848 (54)	7464 (54)	10,294 (54)
eGFR_Cr-CysC_ (ml/min/1.73 m^2^)	90.2 (14.0)	90.1 (14.0)	90.4 (14.2)	90.1 (14.1)	90.0 (14.0)	90.5 (14.0)	90.3 (14.1)	90.2 (14.0)	90.2 (14.0)
eGFR_Cr-CysC_ < 60 ml/min/1.73 m^2^	8538 (2)	3399 (2)	309 (2)	163 (2)	780 (2)	763 (2)	2369 (2)	304 (2)	451 (2)
eGFR_CysC_ (ml/min/1.73 m^2^)	88.2 (16.0)	88.1 (16.0)	88.4 (16.1)	88.2 (16.0)	88.0 (16.0)	88.5 (16.0)	88.3 (16.0)	88.2 (15.9)	88.3 (16.0)
eGFR_CysC_ < 60 ml/min/1.73 m^2^	16,230 (5)	6478 (5%)	594 (5%)	288 (4%)	1474 (5%)	1428 (4%)	4503 (5%)	591 (4%)	874 (4.6%)
ACR (mg/g)	28.1 (135)	27.5 (129)	26.4 (91)	35.2 (255)	27.8 (117)	28.6 (137)	28.6 (136)	24.8 (95)	31.0 (175)
ACR > 30 mg/g	16,021 (14)	6299 (14)	593 (15)	269 (13)	1448 (14)	1473 (14)	4396 (14)	628 (14)	915 (15)

Dichotomous variables are presented as number (percent). Continuous variables are presented as mean (standard deviation)

*ACR* albumin–creatinine ratio, *eGFR*_*Cr-CysC*_ estimated glomerular filtration rate by combined CKD-EPI equation for serum creatinine and cystatin C, *eGFR*_*CysC*_ estimated glomerular filtration rate by CKD-EPI equation for cystatin C

Clinical characteristics were similar across mtDNA haplotypes. The mean (SD) age was 57 ± 8 years and 54% of participants were female. Diabetes mellitus was present in 6% of the cohort, whereas hypertension was prevalent in 54% of participants. The mean eGFR_Cr-CysC_ was 90 ± 14 ml/min/1.73 m^2^ and mean eGFR_CysC_ was 88 ± 16 ml/min/1.73 m^2^. Only 2% of participants had an eGFR_Cr-CysC_ < 60 ml/min/1.73 m^2^, whereas 5% had an eGFR_CysC_ < 60 ml/min/1.73 m^2^. The mean urine ACR was 28 mg/g and 14% of participants had a urine ACR > 30 mg/g.

### Associations of mtDNA haplotypes with CKD and ESKD

MtDNA haplotype was significantly associated with the continuous outcome of eGFR_Cr-CysC_ (Table 2; *p* value for model, 2.8E-12). When compared to the reference haplotype, mtDNA haplotypes I (*β* = 0.402, standard error (SE) = 0.111; *p* = 2.7E−4), IV (*β* = 0.430, SE = 0.073; *p* = 4.2E−9), and V (*β* = 0.233, SE = 0.050; p = 2.7E−6) were each associated with higher eGFR_Cr-CysC_. When we adjusted additionally for diabetes mellitus and hypertension, the association of mtDNA haplotype with eGFR remained robust (*p* = 1.2E−10). In sensitivity analyses, addition of haplogroup, which largely captures mitochondrial genetic variation due to genetic ancestry, as a covariate to a regression model with haplotype did not improve the model (*p* = 0.34). By contrast, addition of haplotype into a model with haplogroup significantly improved the model (*p* = 1.26E−5), indicating the observed associations are driven by haplotype rather than ancestral haplogroup or population substructure.

**Table 2:** Associations of mitochondrial DNA haplotypes with eGFR and risk of CKD (*N* = 362,802)

	Continuous eGFR_Cr-CysC_			eGFR_Cr-CysC_ < 60 ml/min/1.73 m^2^ *N* = 8538 cases	
	*β* Coefficient	Standard error	*p* value	Risk ratio (95% CI)	*p* value
*mtDNA haplotype*					
I	0.402	0.111	2.7E−4	1.00 (0.89, 1.13)	0.95
II	0.122	0.151	0.42	1.05 (0.89, 1.23)	0.59
III	− 0.144	0.074	5.0E−2	1.01 (0.94, 1.10)	0.73
IV	0.430	0.073	4.2E−9	0.98 (0.90, 1.06)	0.59
V	0.233	0.050	2.7E−6	1.00 (0.96, 1.06)	0.74
VI	0.172	0.107	0.11	0.93 (0.83, 1.05)	0.26
VII	0.162	0.093	8.1E−2	1.00 (0.90, 1.10)	0.93
*p* value for model	2.8E−12			0.93	

Model adjusts for age, age squared, sex, center, 40 principal components, and genotyping array

Beta coefficients and risk ratios compare each displayed mtDNA haplotype to the reference mtDNA haplotype

*CKD* chronic kidney disease, *CI* confidence interval, *eGFR* estimated glomerular filtration rate by combined CKD-EPI equation for serum creatinine and cystatin C

In a secondary analyses to identify potential associations not captured by the haplotype analyses, we conducted single SNP analyses among the subset of participants with whole-genome sequencing data available (*N* = 144,741). We tested 3,189 variants with minor allele count ≥ 20, using rank normal transformed eGFR to protect against outlier measures unduly influencing rare variant results (Supplemental Table 3). The most significant association observed for a rare variant was mt16214C > T (*p* < 7.65 × 10^–5^, MAF = 0.001). The most significant variant with MAF ≥ 0.01 was mt3010G > A (*p* < 0.0007, MAF = 0.13), which has previously been associated with eGFR (Yonova-Doing et al. 2021). Neither of these variants passed Bonferroni correction for multiple testing.

**Table 3:** Associations of mitochondrial DNA haplotypes with risk of ESKD

	Prevalent ESKD *N* = 416 cases		Incident ESKD *N* = 405 events	
	Risk ratio (95% CI)	*p* value	Hazard ratio (95% CI)	*p* value
*mtDNA haplotype*				
I	1.22 (0.78, 1.92)	0.39	0.98 (0.56, 1.69)	0.93
II	0.82 (0.39, 1.75)	0.61	1.39 (0.73, 2.64)	0.31
III	0.54 (0.35, 0.83)	5.4E−3	1.11 (0.79, 1.58)	0.54
IV	0.98 (0.70, 1.38)	0.92	0.88 (0.60, 1.29)	0.53
V	0.79 (0.62, 1.00)	5.3E−2	1.01 (0.79, 1.29)	0.93
VI	0.75 (0.43, 1.31)	0.31	1.22 (0.75, 1.98)	0.43
VII	0.85 (0.54, 1.33)	0.47	1.01 (0.64, 1.60)	0.95
P value for model	5.9E−2		0.93	

Model adjusts for age, age squared, sex, center, 40 principal components, and genotyping array

Risk ratios and hazard ratios compare each displayed mtDNA haplotype to the reference mtDNA haplotype

*ESKD* end-stage kidney disease, *CI* confidence interval

Findings were similar when we examined associations of mtDNA haplotype with eGFR_CysC_ (Supplemental Table 4; p value for model, *p* = 2.2E−7). MtDNA haplotypes I (*β*  = 0.351, SE = 0.126; *p* = 5.4E−3), IV (*β* = 0.405, SE = 0.083; *p* = 1.2E−6), and V (*β* = 0.186, SE = 0.056; *p* = 1.0E−3) were each associated with higher eGFR_CysC_ as compared to the reference haplotype. By contrast, mtDNA haplotype was not associated with the dichotomous outcome of eGFR < 60 ml/min/1.73m^2^ when assessed either by creatinine and cystatin C (*p* = 0.93) or by cystatin C alone (*p* = 0.69).

**Table 4:** Associations of mitochondrial DNA haplotypes with risk of AKI (*N* = 379,432)

	AKI *N* = 14,170 cases	
	Risk ratio (95% CI)	*p* value
*mtDNA haplotype*		
I	1.00 (0.91, 1.10)	0.95
II	1.12 (0.99, 1.27)	6.8E−2
III	1.04 (0.98, 1.11)	0.18
IV	0.96 (0.90, 1.03)	0.24
V	1.00 (0.95, 1.04)	0.85
VI	1.05 (0.96, 1.15)	0.29
VII	1.03 (0.95, 1.12)	0.44
P value for model	0.26	

Model adjusts for age, age squared, sex, center, 40 principal components, and genotyping array

Risk ratios compare each displayed mtDNA haplotype to the reference mtDNA haplotype

*AKI* acute kidney injury, *CI* confidence interval

There were 416 cases of ESKD at the baseline assessment, and an additional 405 ESKD events occurred over approximately 12 years of follow-up (Table 3). There were no significant associations of mtDNA haplotype with prevalent or incident ESKD (*p* = 5.9E−2 and *p* = 0.93, respectively).

### Associations of mtDNA haplotypes with AKI and albuminuria

Among the 379,432 UK Biobank participants in this analysis, there were a total of 14,170 cases of AKI with 13,693 occurring after the baseline assessment. There were no significant associations of mtDNA haplotype with risk of AKI (Table 4; p value for model, 0.26). Similarly, there were no significant associations of mtDNA haplotype with urine ACR (*p* = 0.54) or ACR > 30 mg/g (*p* = 0.35) in the overall models (Supplemental Table 5).

## Discussion

The kidney is a metabolically active organ with the highest oxygen consumption rate after the heart (Pagliarini et al. 2008; O'Connor Oct 2006; Wang et al. 2010). Healthy mitochondrial function is, therefore, required for the bioenergetic processes that drive solute transport, elimination of toxins, acid–base homeostasis, and regulation of volume status. As evolutionary descendants of bacterial endosymbionts, mitochondria contain their own circular mtDNA that encodes the translational machinery and proteins critical for oxidative phosphorylation (Andersson et al. 1998; Giles et al. 1980; Anderson et al. 1981). We hypothesized that variability in this maternally inherited genome could contribute to the risk of kidney dysfunction and injury. In this population-based cohort study, we found that mtDNA haplotype is associated with eGFR, but not with risk of ESKD, AKI or albuminuria. The associations of mtDNA haplotype with eGFR remained robust even after adjustment for the presence of diabetes mellitus and hypertension, suggesting the effects of mtDNA haplotype are not driven by mediation through these known risk factors.

Prior studies within the UK Biobank and the Biobank Japan Project reported associations of mtDNA variants with eGFR (Yonova-Doing et al. 2021; Yamamoto et al. 2020). The present study builds upon existing literature by examining associations of mtDNA haplotypes with not only eGFR, but also ESKD, AKI, and albuminuria. We previously found that mtDNA haplotypes are causally associated with mtDNA copy number, a measure of mitochondrial abundance, and several associated traits (Longchamps et al. 2022). Here, we observed independent associations of mtDNA haplotype with eGFR, and compared to the reference haplotype, three mtDNA haplotypes with population fractions of 4%, 9%, and 28%, respectively, had significantly higher eGFR of 0.23–0.43 ml/min/1.73 m^2^ in magnitude. In our prior work, we did not observe patterns that would suggest mediation by other phenotypic traits for the associations of mtDNA haplotypes and kidney function (Longchamps et al. 2022). Adjustment for blood counts, liver enzymes, or mtDNA copy number also did not attenuate associations of the haplotypes with eGFR (data not shown), suggesting the mtDNA genome has direct effects on kidney health. Further studies are needed to determine the functional impact of specific mtDNA haplotypes, including identifying the individual underlying causal variants, and to uncover the mechanisms underlying their associations with kidney risk.

Although we did not detect significant associations of mtDNA haplotype with ESKD risk, possible explanations include our limited power and the potential impact of survival bias, as individuals with CKD often die prior to the development of ESKD. Additionally, our evaluation of common variants may have reduced our ability to detect variants that are under negative selection due to their impact on ESKD risk. The lack of association of mtDNA haplotype with AKI was unexpected because of experimental evidence linking mitochondrial function with both protection from and recovery following AKI (Emma et al. 2016). Despite our large sample size overall, our use of diagnostic codes to identify AKI cases may have reduced accuracy for this outcome. We were unable to use changes in serum creatinine to define AKI due to the unavailability of these data within the UK Biobank database.

Strengths of this study include the large sample size with mtDNA data, our ascertainment of eGFR by both serum creatinine and cystatin C, our inclusion of albuminuria, and our use of an algorithmically defined ESKD outcome. There are important limitations to this study. First, as a study of UK Biobank participants who self-identified as white, our findings may not be generalizable to other populations. Second, the UK Biobank is healthier than the sampling population with evidence of a “healthy volunteer” selection bias (Fry et al. 2017). Hence, the burden of CKD and CKD risk factors was lower than would be expected for the general population, and may have reduced power for the outcome of prevalent CKD. Third, this study was not designed to evaluate changes in eGFR over time. Fourth, we did not have access to the etiology of ESKD or kidney transplant status, so we could not determine whether associations might differ by underlying disease or transplant status.

In conclusion, among UK Biobank participants who self-identified as white, mtDNA haplotype was associated with eGFR, but not with ESKD risk, AKI risk or albuminuria. Compared to the most prevalent haplotype, mtDNA haplotypes I, IV, and V were associated with higher eGFR. Future studies are needed to provide insight into the biological mechanisms underlying these associations.

### Supplementary Information

Below is the link to the electronic supplementary material.

Supplementary file1 (DOCX 23 KB)

Supplementary file2 (XLSX 1067 KB)

## Data Availability

The dataset analyzed in the current study is available from the UK Biobank to approved researchers.
