# Supplementary material for: Mitochondrial genetic variation and risk of chronic kidney disease and acute kidney injury in UK Biobank participants
Source: Hum Genet. 2024 Feb 13;143(2):151–7. doi: 10.1007/s00439-023-02615-4 (PMC10881785; doi:10.1007/s00439-023-02615-4)
Supplement: Supplementary file 1 — Supplementary file1 (DOCX 23 KB) [file 439_2023_2615_MOESM1_ESM.docx]

**Supplemental File: Table of Contents**

Supplemental Table 1: Mitochondrial DNA haplotypes with minor allele frequency >0.5% among UK Biobank participants who self-identified as white

Supplemental Table 2: ICD9 and ICD10 diagnostic codes used to define acute kidney injury (A) and diabetes mellitus (B)

Supplemental Table 4: Associations of mitochondrial DNA haplotypes with eGFR and risk of CKD by cystatin C (N = 362,802)

Supplemental Table 5: Associations of mitochondrial DNA haplotypes with albuminuria (N = 114,662)

**Supplemental Table 1: Mitochondrial DNA haplotypes with minor allele frequency >0.5% among UK Biobank participants who self-identified as white**

| Reference | 2_2_2_2_2_2 |
| --- | --- |
| I | 0_0_0_2_2_2 |
| II | 0_0_2_0_2_0 |
| III | 0_0_2_0_2_2 |
| IV | 0_0_2_2_0_2 |
| V | 0_0_2_2_2_2 |
| VI | 0_2_2_2_2_2 |
| VII | 2_0_2_2_2_2 |

Notes: MtDNA haplotypes are notated in the following format: MT73_MT7028_MT10238_MT12612_MT13617_MT15257
0 indicates revised Cambridge Reference Sequence allele

**Supplemental Table 2: ICD9 and ICD10 diagnostic codes used to define acute kidney injury (A) and diabetes mellitus (B)**

1. **Acute kidney injury, ICD9 and ICD10 diagnostic codes**

| **Type** | **Code** | **Description** |
| --- | --- | --- |
|  |  |  |
|  |  |  |
| ICD9 Diagnostic Codes | 584 | Acute kidney failure |
|  | 584.5 | Acute kidney failure with lesion of tubular necrosis |
|  | 584.6 | Acute kidney failure with lesion of renal cortical necrosis |
|  | 584.7 | Acute kidney failure with lesion of renal medullary necrosis |
|  | 584.8 | Acute kidney failure with other specified pathological lesion in kidney |
|  | 584.9 | Acute kidney failure, unspecified |
| ICD10 Diagnostic Codes | N17.0 | Acute kidney failure with tubular necrosis |
|  | N17.1 | Acute kidney failure with acute cortical necrosis |
|  | N17.2 | Acute kidney failure with medullary necrosis |
|  | N17.8 | Other acute kidney failure |
|  | N17.9 | Acute kidney failure, unspecified |

1. **Diabetes mellitus, ICD9 and ICD10 diagnostic codes**

| **Type** | **Code** | **Description** |
| --- | --- | --- |
|  |  |  |
|  |  |  |
| ICD9 Diagnostic Codes | 250.0 | Diabetes mellitus without mention of complication |
|  | 250.1 | Diabetes with ketoacidosis |
|  | 250.2 | Diabetes with hyperosmolarity |
|  | 250.3 | Diabetes with coma |
|  | 250.4 | Diabetes with renal manifestations |
|  | 250.5 | Diabetes with ophthalmic manifestations |
|  | 250.6 | Diabetes with neurologic manifestations |
|  | 250.7 | Diabetes with peripheral circulatory disorders |
|  | 250.9 | Diabetes with unspecified complication |
| ICD10 Diagnostic Codes | E10 | Insulin-dependent diabetes mellitus |
|  | E11 | Non-insulin-dependent diabetes mellitus |
|  | E13 | Other specified diabetes mellitus |
|  | E14 | Unspecified diabetes mellitus |

**Supplemental Table 4: Associations of mitochondrial DNA haplotypes with eGFR and risk of CKD by cystatin C (N = 362,802)**

|  | **Continuous eGFR_CysC_** | | |  | **eGFR_CysC_<60ml/min/1.73m^2^**  **N = 16,230 cases** | |  |
| --- | --- | --- | --- | --- | --- | --- | --- |
|  | **β Coefficient** | **Standard Error** | **P-Value** |  | **Risk Ratio**  **(95% CI)** | **P-Value** |  |
| ***mtDNA haplotype*** |  |  |  |  |  |  |  |
| I | 0.351 | 0.126 | 5.4E-3 |  | 1.02 (0.93, 1.11) | 0.75 |  |
| II | 0.169 | 0.173 | 0.33 |  | 0.96 (0.85, 1.09) | 0.56 |  |
| III | -0.143 | 0.084 | 8.7E-2 |  | 1.00 (0.94, 1.06) | 0.97 |  |
| IV | 0.405 | 0.083 | 1.2E-6 |  | 0.96 (0.90, 1.02) | 0.15 |  |
| V | 0.186 | 0.056 | 1.0E-3 |  | 1.01 (0.97, 1.05) | 0.71 |  |
| VI | 0.142 | 0.122 | 0.24 |  | 0.95 (0.87, 1.04) | 0.26 |  |
| VII | 0.202 | 0.106 | 5.5E-2 |  | 1.02 (0.94, 1.10) | 0.66 |  |
|  |  |  |  |  |  |  |  |
| ***P-Value for model*** | 2.2E-7 | | |  | 0.69 | |  |
|  |  |  |  |  |  |  |  |

Model adjusts for age, age squared, sex, center, 40 principal components, and genotyping array. Beta coefficients and risk ratios compare each displayed mtDNA haplotype to the reference mtDNA haplotype.

Abbreviation: CKD, chronic kidney disease; CI, confidence interval; eGFR_CysC_, estimated glomerular filtration rate by CKD-EPI equation for serum cystatin C

**Supplemental Table 5: Associations of mitochondrial DNA haplotypes with albuminuria (N = 114,662)**

|  | **Continuous ACR** | | |  | **ACR>30mg/g**  **N = 16, 021 cases** | |  |
| --- | --- | --- | --- | --- | --- | --- | --- |
|  | **β Coefficient** | **Standard Error** | **P-Value** |  | **Risk Ratio**  **(95% CI)** | **P-Value** |  |
| ***mtDNA haplotype*** |  |  |  |  |  |  |  |
| I | 0.015 | 0.016 | 0.34 |  | 1.05 (0.96, 1.15) | 0.28 |  |
| II | 0.009 | 0.022 | 0.69 |  | 0.92 (0.81, 1.05) | 0.23 |  |
| III | -0.002 | 0.011 | 0.84 |  | 1.01 (0.95, 1.07) | 0.79 |  |
| IV | 0.011 | 0.010 | 0.28 |  | 1.01 (0.95, 1.07) | 0.86 |  |
| V | -0.001 | 0.007 | 0.92 |  | 0.99 (0.95, 1.04) | 0.81 |  |
| VI | 0.004 | 0.015 | 0.81 |  | 1.03 (0.94, 1.12) | 0.53 |  |
| VII | 0.026 | 0.013 | 5.2E-2 |  | 1.08 (1.00, 1.17) | 3.7E-2 |  |
|  |  |  |  |  |  |  |  |
| ***P-Value for model*** | 0.54 | | |  | 0.35 | |  |
|  |  |  |  |  |  |  |  |

Model adjusts for age, age squared, sex, center, 40 principal components, and genotyping array. Beta coefficients and risk ratios compare each displayed mtDNA haplotype to the reference mtDNA haplotype. Continuous ACR is log-transformed.

Abbreviations: ACR, albumin-creatinine ratio; CI, confidence interval
